# Does Gender Moderate the Relationship Between Chronic Pain and Substance Use Disorder? Insights From a National Canadian Population Survey

**DOI:** 10.3389/fpsyt.2022.799655

**Published:** 2022-03-02

**Authors:** Yingying Su, Xiangfei Meng, Carl D'Arcy

**Affiliations:** ^1^School of Public Health, University of Saskatchewan, Saskatoon, SK, Canada; ^2^Department of Psychiatry, McGill, University and the Douglas Research Centre, Montreal, QC, Canada; ^3^Department of Psychiatry, University of Saskatchewan, Saskatoon, SK, Canada

**Keywords:** substance use disorder, chronic pain, epidemiological, gender, CCHS

## Abstract

**Background:**

Though it has been shown that men have a higher lifetime prevalence of substance use disorder and a lower prevalence of chronic pain than women, there is little research to date focusing on gender differences in the relationship between chronic pain and substance use disorder. This study examined whether gender moderates the relationship of chronic pain and substance use disorder. We also sought to examine the gender differences in the associations between specific pain types—arthritis, migraine, and back pain, and substance use disorder.

**Methods:**

The data were drawn from the 2012 Canadian Community Health Survey-Mental Health (CCHS-MH 2012) with individuals aged 20 years and older living in the 10 Canadian provinces (*N* = 23,089). A two-level logistic mixed effects model was used to account for provincial differences.

**Results:**

Our findings indicated gender moderated the association between arthritis as well as migraine, and substance use disorder. However, no moderation effect of gender on the relationship between back pain and substance use disorder was found. Specifically, the strength of the association between arthritis and substance use disorder was stronger among men (OR_interaction_ = 0.62, 95% CI: 0.53 to 0.73), whereas the strength of the association between migraine and substance use disorder was stronger among women (OR_interaction_ = 1.45, 95% CI: 1.18 to 1.79). In addition, geographical location was found to explain a small proportion (2.3%-2.4%) of the overall variance in SUD.

**Conclusions:**

The results suggest that gender moderated the relations between arthritis as well as migraine, and substance use disorder, respectively. Treatment programs for pain and substance misuse might benefit from an approach tailored to gender differences.

## Introduction

Substance use disorder (SUD) is a global public health problem, especially in high-income countries (1). It poses significant challenges to public health and imposes substantial societal and economic burden having both a direct and indirect impact (2). The United Nations International Narcotics Control Board reported that in North America approximately one in every 20 deaths of individuals aged 15 to 64 years was related to substance misuse (3). In Canada, ~4.4% of the general population is diagnosed with a 12-month SUD, while 21.6% meet the criteria for a SUD during their lifetime (4). Alcohol use disorder (AUD) is the most common subtype of SUD in Canada, but recently opioid use disorder-related deaths continue to rise (5).

Chronic pain has been positively associated with substance use disorder (6, 7). Individuals with pain may be strongly motivated to discount the long-term risks of addiction in an urgent effort to suppress or relieve their pain (8). Dopamine release can be activated by both pain and opioids suggesting that they may share some common neurobiological mechanisms in the brain reward and motivational systems (9). Based on the extension of the “central sensitization” theory, chronic pain patients with nociceptive hypersensitivity can exhibit alcohol/drug craving and relapse via alterations in synaptic plasticity (10). A randomized controlled trial demonstrated that a short-term morphine exposure decreased the pain and altered the reward-related networks among patients with chronic low back pain (11). It is well-supported in the literature that individuals with higher levels of pain intensity have higher levels of consumption alcohol. Alcohol is consumed to cope with stress and unpleasantness and to alleviate the pain-related negative effects (12–14). Furthermore, there is a significant gender difference in this finding, men in contrast to women, tend to report associations between numerous painful medical conditions and frequent drinking problems (15). However, longitudinal evidence indicates that the heaviest drinkers were most likely to decrease their alcohol consumption so these associations may become weaker over time (16).

It is suggested that gender may be an important factor accounting for the significant findings regarding the relation between chronic pain and SUD. While the term “sex” focuses on the biological differences between females and males, “gender” is defined as socially determined roles varying across cultures over time (17). Although increased attention has focused on the importance of gender in the health and well-being over the past 20 years (18), most of prior research has focused on gender as a main effect predictor of pain or substance misuse issues. Recent research has brought forward attention to gender differences in SUD and chronic pain. Men consistently report higher prevalence of use, abuse and dependence as compared to women, for example, nicotine, alcohol and illicit drugs (19, 20). Variations across cultures and policies substantially influence the access to and acceptability of substances use which appears to account for such gender difference in the prevalence of SUD (17). Noticeably, such gender gap in substance use disorders appears to be narrowing (17). In contrast, women are more likely to report chronic pain conditions (21). Men and women react to pain differently due to different societal roles governing their behavior (22).

However, limited research has been conducted to explore the effect of individual types of chronic pain as most studies have clustered different types of chronic pain into one general pain category. There were also very little data characterizing the potential moderating effect of gender on the relationship between specific pain and SUDs. Does the association between pain and substance use disorder vary by gender? There are difficulties in drawing firm conclusions about different gender vulnerability to substance use disorder among those with chronic pain. While one study found that the strength of association between pain and substance use disorder was stronger for women compared to men among college students (23), another study found men with the chronic pain conditions more frequently developed substance misuse (for example, opioid abuse and dependence) than women (24). To fully understand the relationship of pain experiences and substance use disorder, we have to consider how gender relates to the presence of pain, as well as how gender relates to pain-related problems.

Previous studies have indicated that the prevalence of SUD and related problems vary across Canada, with a lower level being found in Atlantic Canada and a higher level in the western provinces (25). Though these studies provide preliminary information on the geographical distribution of substance misuse problems in Canada, no study has addressed the potential explanations underlying those differences. It remains unclear whether the variations in substance use disorder prevalence result from provincial variations in the geographic distribution of pain. Few studies have investigated the moderation effect of gender on the relationship between pain and substance use disorder while taking into consideration spatial lag and spatial clustering, thus a multilevel structure has been suggested. Identifying such geographic variation will be an important step in tailoring priorities for resource allocations to programs aimed at SUD prevention for men and women. From a public policy perspective, it is useful to have such estimates of the impacts of SUD to prioritize allocation of public resources and inform policy in order to promote population health among specific subgroups of the population.

Our current study aims to investigate whether gender moderates the relationship of chronic pain and substance use disorders. We also aim to examine provincial differences in the SUD in Canada. We hypothesized that the association between different types of chronic pain and SUD will differ between men and women and that provincial variations exist in the association between chronic pain and SUD while taking covariates into account.

## Methods

### Data and Sample

Data used to examine these hypotheses are from the Public Use Microdata File (PUMF) of the Canadian Community Health Survey-Mental Health (CCHS-MH 2012), a national cross-sectional population-based omnibus health survey with an emphasis on mental health and healthcare services use. CCHS-MH 2012 is at this time the most recent survey devoted to exploring on the mental health status and behaviors of Canadians. The survey was conducted by Statistics Canada. It targeted household residents 15 years of age and older living in the 10 provinces and 3 territories of Canada. Sampling excluded those living on First Nations reserves and other Indigenous settlements, full-time members of the Canadian Armed Forces and the Royal Canadian Mounted Police (RCMP) as well as residents of commercial, institutional, or communal dwellings that provide care and/or custody services. It is estimated these exclusions account for 3% of the national population. A multistage stratified cluster design based on the monthly national Labour Force Survey was used to ensure adequate coverage by gender and age for each province and territory. A total of 25,113 participants were interviewed using computer assisted personal interviewing (CAPI) and telephone interviewing (CATI) technology. Participation in the survey was voluntary. A total of 87.0% of interviews were conducted in person at participant's home. The combined individual- and household-level response rate was 68.9% (26). Given our interest to understand chronic pain among adults, we restricted our study sample to respondents 20 years of age or older. As a result, this present study included a total of 23,089 individuals, 10,373 men and 12,716 women.

The PUMF for the CCHS-MH 2012 contains anonymized survey data devoid of any personally identifying information. Participants in the original survey signed an informed consent and voluntarily participated in the survey. The original survey received ethical approval through Statistics Canada procedures. Survey sample weights were provided by Statistics Canada to estimate population's prevalence. The secondary analysis of anonymized data does not require ethical approval.

### Measures

#### Lifetime Substance Use Disorder (SUD-LT)

Substance use disorder was measured using the World Health Organization version of Composite International Diagnostic Interview (WHO-CIDI). WHO-CIDI is a structured diagnostic interview that generates diagnosis according to the Diagnostic and Statistical Manual of Mental Disorders, Fourth Edition (DSM-IV) and the International Classification of Disease (ICD-10) (27). *Substance use disorder* was derived from computer-based lifetime algorithms for “Alcohol Abuse or Dependence” and “Drug Abuse or Dependence (including Cannabis)” which were both used to calculate the presence of lifetime substance use disorder (SUD-LT). Since we used the Public Use Microdata File (PUMF) of the Canadian Community Health Survey-Mental Health (CCHS-MH), only SUD-LT was available for examination in the current study.

#### Chronic Pain Condition

Respondents were assessed for 12 different chronic conditions with a group of questions pertaining to long-term health. Respondents were classified as having a chronic condition if they self-reported they had chronic conditions that had already lasted 6 months or more, and had been diagnosed by a health professional. These chronic conditions were grouped into two broad categories in general: physical conditions (i.e., high blood pressure, diabetes, heart disease, etc.) and pain conditions (i.e., arthritis, back pain, and migraine headaches). Those three chronic pain conditions including arthritis (excluding fibromyalgia), back problems (excluding fibromyalgia and arthritis), and migraine headaches were included in our study.

#### Covariates

To assess the inclusion of potential covariates in the model, we examined the correlations between socio-demographic characteristics including age, education, marital status, annual household income, ethnicity and immigrant status and SUD. They were all found to be significantly correlated with substance use disorder, thus were included in the models as covariates (Supplementary Table 1). Respondents were asked standard questions concerning education (Less than secondary, Secondary school graduation, Some post-secondary graduation and Post-secondary graduation), ethnicity (White/Non-white) and immigrant status (Yes/No). For these variables original survey responses were used. Sixteen age categories were reported in the PUMF and they were re-categorized as follows: 20–24, 25–44, 45–64, and 65+ years. Marital status was re-categorized as: single, married/common law, and divorced/separated/widowed. Finally, annual household income was grouped into: Less than $20,000, $20,000–$39,999, $40,000–$59,999 $60,000–$79,999, and $80,000+ for better characterizing the distribution of the income.

## Statistical Analysis

**Figure 1:**
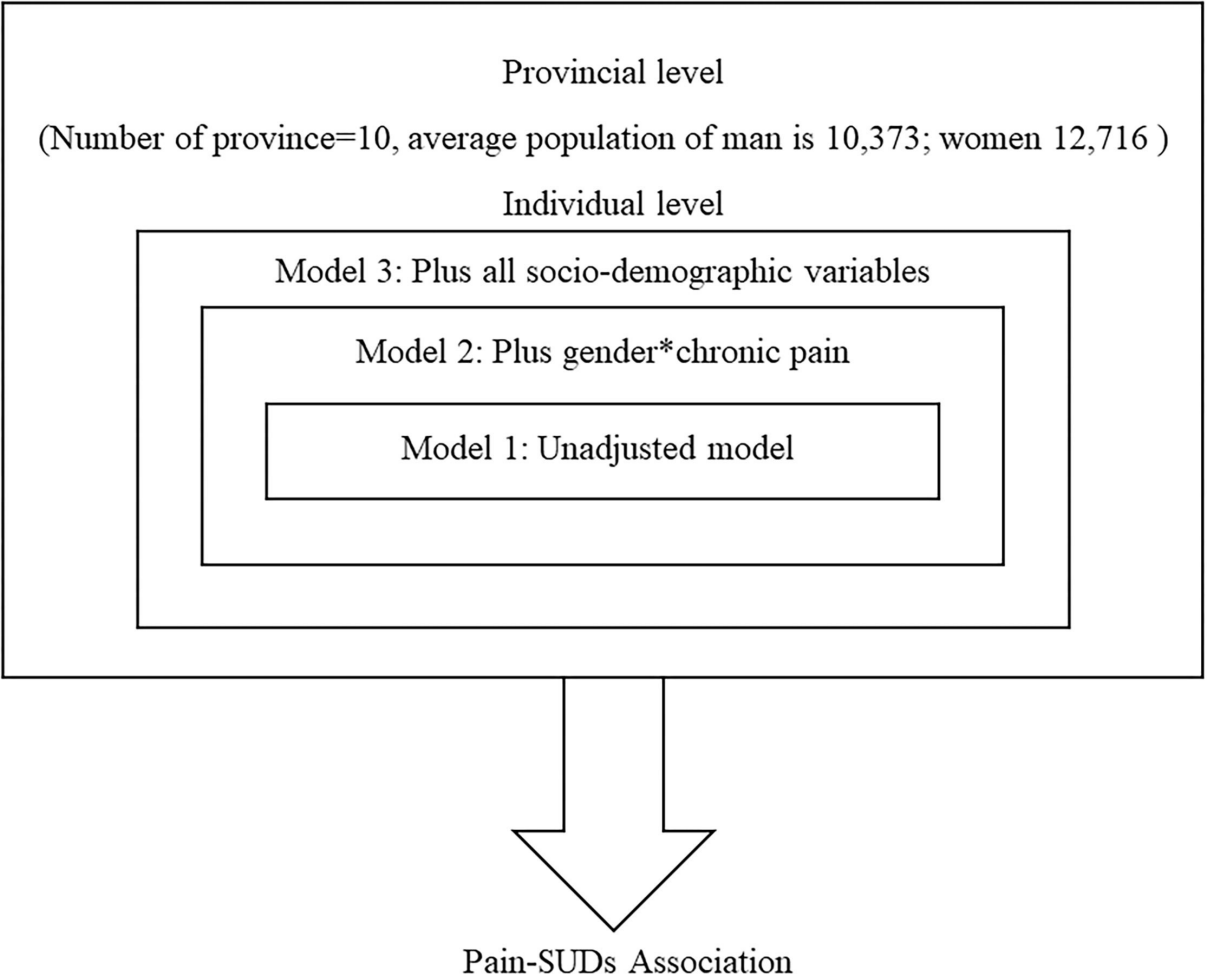
Theoretical-hierarchical model.

Weighted percentages for categorical variables were used to describe the distribution of the respondents by types of chronic pain. We used the Pearson's chi-squared test to examine the differences between three types of chronic pain and sociodemographic characteristics. To consider provincial differences in these relationships, multivariate mixed effects logistic regression was used to explore the association between chronic pain and substance use disorder. The conceptual-hierarchical model which was proposed by Victora et al. (28) and shown in Figure 1 was used as a guide for building the three models. This model involves the partial pooling, thereby addressing the multiple comparisons problems and yielding more efficient estimates (29). The three models were constructed separately controlling for different sets of potential confounders. The first model was an unadjusted model with only chronic pain included. The second model added an interaction term of gender and chronic pain. The final model further included all the selected sociodemographic covariates.

Fixed effects: The results of fixed effects, measure of association between the chronic pain and substance use disorder, were presented as odds ratios (ORs) with corresponding 95% confidence intervals (CIs).

Random effects: The results of random effects which captured the measures of variation, were presented as variance partition coefficients. A random intercept variance component model is fit as a baseline for comparison with the adjusted model. This model shows whether observations are dependent within provinces and are indicative of variations in substance use disorder prevalence between provinces. Intra-class correlation coefficient (ICC) with the statistical formula of: $\rho =\frac{\sigma 2group}{\sigma 2group+\frac{\pi 2}{3}}$ was applied to quantify the proportion of variance in substance use disorder that is attributable to province of residence (30).

To determine whether gender modified this relationship, an interaction term, gender^*^pain was added to the second and the final models for arthritis, migraine and back pains, respectively. Interactions with *p*-values < 0.05 were regarded as significant. Additionally, a likelihood ratio test was then used to compare the goodness of fit of the model without the interaction term to the final model with the interaction term. Simple slopes were generated to determine the association between chronic pain and substance use disorder (with significance) at gender stratification by fitting a model with the main effects of pain and gender and their interaction term.

Because the three models are nested models, model fit was evaluated by comparing the difference in deviance (−2^*^log-likelihood) which is more appropriate for the comparison of nested hierarchical linear models by incorporating the deviance statistic (31). Smaller values for deviance tests indicate a better fit model.

Missing data were imputed with multiple imputation procedure (*m* = 5), with missing data ranging from 0.1 to 0.6%. The recommended procedure of multiple imputation for imputing missing data with regression switching by chained equations was used (32). Multiple imputation has been shown to be valid in reducing bias as long as suitable auxiliary measures were applied to the imputation model (33). All statistical analyses were conducted in Stata version 15.0 (StataCorp LP, College Station, TX). *P*-value of 0.05 was used to define statistical significance.

## Results

### Sociodemographic Characteristics by Different Types of Chronic Pain

Table 1 summarizes the comparisons of sociodemographic characteristics for the three types of chronic pain among the study sample. Differences were observed for all the sociodemographic characteristics. Women reported chronic pain more frequently than men. Respondents aged 25 to 44 years had the highest prevalence of migraine, while those respondents aged 65+ tended to report more back pain and arthritis. Chronic pain was more common among respondents who were divorced, separated, and widowed than respondents who were married or in a common-law relationship. Those with a lower level of education and lower household income reported higher prevalence of chronic pain. Survey respondents who identified themselves as white and non-immigrants tended to report more chronic pain.

**Table 1:** Socio-demographic characteristics, types of chronic pain, Canadian Community Health Survey of Mental Health, 2012 (*N* = 23,089).

**Characteristics**	**Total sample (weighted %)**	**Arthritis (weighted %)**			**Migraine (weighted %)**			**Back Pain (weighted %)**		
	**Percentage**	**Yes**	**No**	*P*-value^*^	**Yes**	**No**	*P*-value^*^	**Yes**	**No**	*P*-value^*^
Gender			<0.001			<0.001			0.001	
Men	49.1%	14.0%	86.0%		6.3%	93.7%		17.9%	82.1%	
Women	50.9%	22.1%	77.9%		14.4%	85.6%		20.2%	79.8%	
Age(years)				<0.001			<0.001			<0.001
20–24	8.3%	1.0%	99.0%		10.8%	89.2%		8.4%	91.6%	
25–44	35.7%	5.2%	94.8%		13.1%	86.9%		15.3%	84.7%	
45–64	37.3%	21.5%	78.5%		10.4%	89.6%		21.5%	78.5%	
65+	18.7%	43.6%	56.4%		5.2%	94.8%		26.3%	73.7%	
Marital status				<0.001			<0.001			<0.001
Single	20.7%	7.5%	92.5%		10.6%	89.4%		14.8%	85.2%	
Married/Common law	65.3%	18.3%	81.7%		10.1%	89.9%		19.2%	80.8%	
Divorced/Separated/Widowed	14.%	32.9%	67.1%		11.3%	88.7%		24.5%	75.5%	
Education level				<0.001			<0.001			<0.001
Less than secondary	14.8%	30.9%	69.1%		10.5%	89.5%		23.9%	76.1%	
Secondary school graduation	15.6%	17.8%	82.2%		10.3%	89.7%		19.1%	80.9%	
Some post-secondary graduation	5.9%	16.0%	84.0%		11.0%	89.0%		17.5%	82.5%	
Post-secondary graduation	63.7%	15.4%	84.6%		10.4%	89.6%		18.1%	81.9%	
Annual household income				<0.001			<0.001			<0.001
Less than $20,000	4.5%	28.7%	71.3%		14.0%	86.0%		30.7%	69.3%	
$20,000–$39,999	12.5%	29.9%	70.1%		12.0%	88.0%		26.3%	73.7%	
$40,000–$59,999	18.5%	22.6%	77.4%		10.3%	89.7%		19.6%	80.4%	
$60,000–$79,999	17.6%	19.1%	80.9%		10.5%	89.5%		18.3%	81.7%	
$80,000+	46.9%	11.8%	88.2%		9.6%	90.4%		16.2%	83.8%	
Ethnicity				<0.001			<0.001			0.001
White	77.5%	20.3%	79.7%		10.8%	89.2%		20.3%	79.7%	
Non-white	22.5%	10.6%	89.4%		9.1%	90.9%		14.9%	85.1%	
Immigrant				<0.001			<0.001			<0.001
Yes	26.5%	15.6%	84.4%		7.8%	92.2%		16.3%	83.7%	
No	73.5%	19.0%	81.0%		11.3%	88.7%		20.0%	80.0%	

* *Indicates p-value is from chi-square tests*.

### How Gender and Chronic Pain Interact to Impact Substance Use Disorder With Covariates Adjustment

Tables 2–4 present the findings of the main effects of different types of chronic pain and interactive effects of gender and each type of chronic pain (Gender × Pain conditions) on the SUD. Three multilevel regression models were employed to examine whether gender moderated the relationship between chronic pain and SUD with or without adjustment of the selected sociodemographic variables.

**Table 2:** Two-level univariate/multivariate models with interaction terms measuring association between arthritis and lifetime substance use disorder, Canadian Community Health Survey of Mental Health, 2012 (*N* = 23,089).

**Variables**	**Odds ratios (95% Confidence Interval)**		
	**Model 1**	**Model 2**	**Model 3**
Chronic pain			
Yes	**0.82 (0.77, 0.89)**	**1.20 (1.08, 1.33)**	**1.54 (1.37, 1.72)**
No	1	1	1
Gender			
Women		**0.33 (0.31, 0.35)**	**0.31 (0.28, 0.33)**
Men		1	1
Gender × Chronic pain			
Women × arthritis		**0.20 (0.18, 0.23)**	**0.62 (0.53, 0.73)**
Age (years)			
20–24			1
25–44			**1.20 (1.06, 1.36)**
45–64			1.01(0.88, 1.15)
65+			**0.43(0.37, 0.50)**
Marital status			
Single			**1.31 (1.20, 1.43)**
Divorced/Separated/ Widowed			**1.19 (1.08, 1.30)**
Married/Common law			1
Education level			
Less than secondary			**1.10 (1.00,1.21)**
Secondary school graduation			**1.09 (1.00,1.15)**
Some post-secondary graduation			**1.48 (1.29,1.69)**
Post-secondary graduation			1
Annual household income			
Less than $20,000			**1.50 (1.31, 1.73)**
$20,000-$39,999			**1.12 (1.01, 1.25)**
$40,000-$59,999			1.04 (0.95,1.14)
$60,000-$79,999			0.97 (0.88,1.09)
$80,000+			1
Ethnicity			
White			**1.16 (1.04, 1.30)**
Non-white			1
Immigrant			
Yes			**0.32 (0.28, 0.36)**
No			1
Model fit statistics			
−2 Log Likelihood	25,476	23,966	22,718

*Model 1 = unadjusted model; Model 2 = in addition to the interaction term; Model 3 = controlling for sociodemographic (i.e., gender, age, ethnicity/race, household income, education, marital status). Significant odds ratios and 95% confidence intervals are highlighted*.

**Table 3:** Two-level univariate/multivariate models with interaction terms measuring association between migraine and lifetime substance use disorder, Canadian Community Health Survey of Mental Health, 2012 (*N* = 23,089).

**Variables**	**Odds ratios (95% Confidence Interval)**		
	**Model 1**	**Model 2**	**Model 3**
Chronic pain			
Yes	**1.22 (1.11, 1.34)**	**1.30 (1.10, 1.52)**	1.09 (0.92, 1.29)
No	1	1	1
Gender			
Women		**0.27 (0.25, 0.29)**	**0.26 (0.24, 0.28)**
Men		1	1
Gender × Chronic pain			
Women × migraine		**1.45 (1.18, 1.78)**	**1.45 (1.18, 1.79)**
Age (years)			
20–24			1
25–44			**1.20 (1.06, 1.36)**
45–64			1.05 (0.92, 1.20)
65+			**0.48 (0.41, 0.56)**
Marital status			
Single			**1.29 (1.18, 1.41)**
Divorced/Separated/ Widowed			**1.17 (1.07, 1.29)**
Married/Common law			1
Education level			
Less than secondary			**1.10 (1.00, 1.21)**
Secondary school graduation			1.08 (0.99, 1.18)
Some post-secondary graduation			**1.49 (1.31, 1.71)**
Post-secondary graduation			1
Annual household income			
Less than $20,000			**1.51 (1.31, 1.74)**
$20,000–$39,999			**1.14 (1.02, 1.27)**
$40,000–$59,999			1.05 (0.96, 1.16)
$60,000–$79,999			0.97 (0.88, 1.07)
$80,000+			1
Ethnicity			
White			**1.16 (1.04, 1.29)**
Non-white			1
Immigrant			
Yes			**0.32 (0.28, 0.36)**
No			1
Model fit statistics			
−2 Log Likelihood	25,488	23,904	22,728

*Model 1 = unadjusted model; Model 2 = in addition to the interaction term; Model 3 = controlling for sociodemographic (i.e., gender, age, ethnicity/race, household income, education, marital status). Significant odds ratios and 95% confidence intervals are highlighted*.

**Table 4:** Two-level univariate/multivariate models with interaction terms measuring association between back pain and lifetime substance use disorder, the Canadian Community Health Survey of Mental Health, 2012 (*N* = 23,089).

**Variables**	**Odds ratios (95% Confidence Interval)**		
	**Model 1**	**Model 2**	**Model 3**
Chronic pain			
Yes	**1.45 (1.36, 1.54)**	**1.58 (1.44, 1.74)**	**1.63 (1.47, 1.80)**
No	1	1	1
Gender			
Women		**0.30 (0.27, 0.32)**	**0.28 (0.26, 0.30)**
Men		1	1
Gender × Chronic pain			
Women × back pain		0.93 (0.80, 1.08)	0.93 (0.80, 1.08)
Age (years)			
20–24			1
25–44			**1.16 (1.02, 1.32)**
45–64			0.98 (0.86, 1.11)
65+			**0.43 (0.37, 0.51)**
Marital status			
Single			**1.31 (1.20, 1.43)**
Divorced/Separated/ Widowed			**1.16 (1.06, 1.28)**
Married/Common law			1
Education level			
Less than secondary			1.09 (0.99, 1.21)
Secondary school graduation			**1.09 (1.00, 1.19)**
Some post-secondary graduation			**1.48 (1.29, 1.69)**
Post-secondary graduation			1
Annual household income			
Less than $20,000			**1.45 (1.26, 1.67)**
$20,000–$39,999			1.11 (0.99, 1.19)
$40,000–$59,999			1.04 (0.95, 1.14)
$60,000–$79,999			0.97 (0.88, 1.07)
$80,000+			1
Ethnicity			
White			**1.17 (1.05, 1.30)**
Non-white			1
Immigrant			
Yes			**0.32 (0.29, 0.36)**
No			1
Model fit statistics			
−2 Log Likelihood	31,158	23,872	22,642

*Model 1 = unadjusted model; Model 2 = in addition to the interaction term; Model 3 = controlling for sociodemographic (i.e., gender, age, ethnicity/race, household income, education, marital status). Significant odds ratios and 95% confidence intervals are highlighted*.

#### Arthritis and Substance Use Disorder

After fully controlling for social-demographic variables in the final model, arthritis was significantly associated with substance use disorder (OR = 1.54, 95% CI: 1.37 to 1.72). We also identified statistically significant interactions between gender and arthritis (OR_Gender × *Arthritis*_ = 0.62, 95% CI: 0.53 to 0.73), suggesting a stronger association between arthritis and SUD among men compared to women.

An examination of the simple slopes confirmed that the interaction effects identified were consistent with the findings of the two-level regression analysis. This gender stratified analysis indicated that the direction of the association between arthritis and SUD varied across gender groups. The link between arthritis and SUD was substantially stronger among men compared to women. Table 5 presented all pairwise comparisons between gender and subtypes of chronic pain.

**Table 5:** A pairwise comparison of the interaction between gender and chronic pain with its influence on lifetime substance use disorder in the Canadian Community Health Survey of Mental Health, 2012 (*N* = 23,089).

**Comparison**	**Arthritis**		**Migraine**		**Back pain**	
	**OR (95% CI)**	***p*-value**	**OR (95% CI)**	***p*-value**	**OR (95% CI)**	***p*-value**
Women-No pain vs. Men-No pain	0.33 (0.31, 0.35)	<0.001	0.26 (0.24, 0.28)	<0.001	0.28 (0.26, 0.30)	<0.001
Men-With pain vs. Men-No pain	1.20 (1.08, 1.33)	<0.001	1.10 (1.00, 1.29)	<0.001	1.63 (1.47, 1.80)	<0.001
Women-With pain vs. Men-No pain	0.25 (0.22, 0.27)	<0.001	0.41 (0.36, 0.47)	<0.001	0.43 (0.38, 0.48)	<0.001
Men-With pain vs. Women-No pain	3.66 (3.28, 4.08)	<0.001	4.19 (3.53, 4.97)	<0.001	5.77 (5.18, 6.43)	<0.001
Women-With pain vs. Women-No pain	0.75 (0.67, 0.84)	<0.001	1.58 (1.39, 1.80)	<0.001	1.51 (1.35, 1.70)	<0.001
Women-With pain vs. Men-With pain	0.20 (0.18, 0.23)	<0.001	0.38 (0.31, 0.46)	<0.001	0.26 (0.23, 0.30)	<0.001

#### Migraine and Substance Use Disorder

Univariable analyses show that migraine was positively associated with SUD. However, after fully controlling for social-demographic variables, migraine was no longer associated with SUD (OR = 1.09, 95% CI: 0.92 to 1.29). The interactive effect of gender and migraine was significant on SUD (OR_Gender × *Migraine*_ = 1.45, 95% CI: 1.18 to 1.79) which indicates that women show stronger association between migraine and SUD than men. The result findings on the pairwise comparison of the interaction between gender and migraine with its influence on substance abuse showed that migraine was associated with SUD for both genders.

#### Back Pain and Substance Use Disorder

Likewise, univariable analyses show that back pain was positively associated with SUD in the univariable analyses. And this positive association remained significant after potential covariates were controlled (OR = 1.63, 95% CI: 1.47 to 1.80). However, interaction between back pain and gender was not associated with SUD (OR_Gender × *Backpain*_ = 0.93, 95% CI: 0.80 to 1.08).

### Provincial Variations in the Association Between Pain Experiences and SUD

The final model showed significant between-province variations of SUD, the intra-class correlation was 0.024 for arthritis, 0.024 for migraine and 0.023 for back pain, separately, indicating that 2.4, 2.4, and 2.3% SUD variation can be attributed to differences between different provinces. Additionally, a comparison of the models using deviance tests showed that the final model had a better fit with smaller values of −2 Log Likelihood compared to other models for all three subtypes of chronic pain.

## Discussion

### Summary of Findings

Using data from a large, representative study of the Canadian population, the present study examined the moderation effect of gender on the association between chronic pain and SUD among Canadian adults. Women reported a higher prevalence of chronic pain compared to men, while substance use disorder was far less common in women compared to men. Our findings also suggest that the effect of arthritis as well as migraine on the SUD was different between men and women. The strength of the association between arthritis and substance use disorder was stronger among men, whereas the strength of the association between migraine and substance use disorder was stronger among women. In addition, the geographical location (province of residence) was found to explain a small proportion (2.3–2.4%) of the overall variance in SUD.

Since the chronic pain conditions were self-reported if “they were diagnosed by a health professional,” this may have resulted in an underestimation of the prevalence of chronic pain among some subpopulation groups, such as non-white people and immigrants who are more likely to be disadvantaged in access to healthcare services (34). Our study findings are consistent with previous studies reporting of a higher prevalence of chronic pain conditions but lower prevalence of substance use disorder among women compared to men (17, 35). These trends suggest that gender differences in the prevalence of both SUD and chronic pain may be attributable to other psychosocial factors, such as social and cultural difference including violence exposures, tolerance level, social support and coping strategies (17, 36). Gender differences in pain have been attributed to a different socialization process for men and women (37). Social roles for women are more supportive of pain expression and awareness, making them a greater acceptance to disclose pain or engage in pain-related behaviors (38). It has also been reported that women with chronic pain were more likely to use emotion-focused coping strategies than men, such as seeking emotional support. This is beneficial in reducing the negative impact of pain and preventing from substances use (36).

### Arthritis and Substance Use Disorder

Our findings confirm that gender plays a moderating role in the link between arthritis and SUD while controlling for other confounding variables which may potentially mask this relationship. Men with arthritis consistently report an elevated prevalence of SUD compared to women in the current study. There is agreement that there are differences in the progression of arthritis between women and men which might be due to the changes in the hormonal state of women in certain life stages (39). It has been reported that men were diagnosed earlier than women and had a more aggressive course (40, 41). The significant functional limitations and disability attend with arthritis may account for this (42). Additionally, dysfunctional beliefs such as hopelessness and loss of self-worth in face of the chronic pain contribute to adverse mental health outcomes (43). Differences in the social stressors, coping resources and opportunity structures could account for the potential moderating effect of gender (44). It has been reported that men with arthritis are more likely to misuse substances to cope with social and behavioral problems or engender a pleasant mood. Recreational substance use may in turn relieve the symptoms of pain without that being the primary intention (45).

The present study also suggests a reverse pattern for men and women in the relationship between arthritis and SUD. We found that men have a higher probability of SUD which is independent of arthritis status when factors associated with both chronic pain and SUD were taken into account. However, in the group of women without arthritis, the prevalence of SUD is much lower than in the group of women with arthritis. There are plausible explanations for this finding. It has been suggested that the substance use disorder, including illegal drug usage and alcohol abuse/dependence by women is indicative of maladaptive behaviors contrary to cultural and social norms, and may result in stigma and social discrimination (46, 47). Controlled substances could be either a problem or a solution under different circumstances; this should dictate a change in optimal treatment depending on individual circumstances (48).

### Migraine and Substance Use Disorder

We found that the gender × migraine interaction was statistically significant, this is evidence for a gender moderation effect, suggesting that women with chronic migraines have higher possibility of endorsing a lifetime substance use disorder as compared to men. Such a gender influence on the association between migraine and substance use disorder is consistent with a population-based prospective cohort study on the headache of adolescents. The findings demonstrated that women with migraine appear to be more susceptible to the substance use disorder (49). Migraine was more likely to be diagnosed and treated in women compared with men. In addition, men with migraine generally have less severe attacks and a lower attack frequency, and less disability which may account for this phenomenon (50).

### Back Pain and Substance Use Disorder

As expected, our study supports epidemiological and clinical research findings that individuals with back pain were more likely to continuously report positive associations with SUD across the life span (51–53). There is no evidence of gender differences in the relationships between back pain and adult SUD in the present study. Men and women with back pain have similar SUD prevalence suggesting that men and women have equivalent odds of having a SUD. Back pain is a complex condition with several factors contributing to its occurrence including physical factors, social demographic characteristics and psychological factors (50). Gender difference in help-seeking for those with chronic pain does not shed light on our findings as evidence shows that the assumption of greater consultation amongst women for back pain is surprisingly weak and inconsistent (54).

### Provincial Variations in the Association Between Pain Experiences and SUD

Little variation in SUD attributable to province of residence was found. The limited variation between provinces that we found in the present study is more likely due to the nature of association of pain and SUD. Even though a previous study of SUD prevalence showed substantial geographical variability at both provincial and municipal levels in Canada (55), differences in the underlying prevalence of SUD are unlikely explanations for the differences in the pain-SUD associations. Our findings do not mean that provincial context and provincial environments do not play an important role in substance misuse patterns for their residents. Unlike our results, geographical differences in chronic pain prevalence, pain intensity and substance misuse have been observed in another jurisdiction—England (56). Future research examining provincial- or municipal-level risk factors could help targeting and making prevention more locally relevant.

The current findings have several implications for public health prevention as well as clinical practice. Our study suggests that health policy makers could gain insight by carefully evaluating individuals with chronic pain when developing a comprehensive prevention strategy to mitigate the onset of SUD. In addition, since the associations between different types of chronic pain and SUD differ among men and women, general practitioners treating individuals for different types of pain should consider integrated collaborative chronic pain interventions for the prevention of SUD in a gender-informed manner.

### Strengths and Limitations

To our knowledge, this is a first study examining gender differences in the relationship between specific types of chronic pain and SUD using data from a large nationally representative survey. We report novel findings that SUD associated with chronic pain differ based on person's gender. These findings shed new light on how chronic pain is linked to SUD. The present study also explored provincial-level differences in SUD across Canada with the intention of guiding future research to investigate contextual factors related to prevention rather than treatment.

Several limitations should be acknowledged. First, the nature of cross-sectional study limits the causal inference. Based on a strong theoretical framework we only explored the hypothesized model in the present study. Our study highlights the importance of interpreting these results with caution and we suggest future longitudinal research to investigate further on these causal pathways and moderation effects. Second, although the sampling frame of the CCHS is representative of 97% of the Canadian population, exclusion of Canadians living on First nation Reserves and residing in institutions could impact the generalizability of our findings, especially with regards to capturing more diverse samples. Third, chronic pain was ascertained by self-report which could not be validated and may introduce the measurement bias. However, high agreement has been observed from somatic diseases based on self-reported and physician-diagnosed physical conditions from national health data (57). In addition, the diagnosis for chronic pain in the current study heavily relied on whether the participants were willing to seek care from healthcare professionals. We cannot rule out the possibility that there was a proportion of population who did not meet the study criteria for chronic pain in the present study, despite possibly having chronic pain. Thus, the prevalence of the chronic pain might be underestimated. Finally, this study limited its measure of specific types of SUD, future study should expand on this body of knowledge by differentiating from the alcohol dependence or abuse from drug abuse/dependence, and various types of drugs.

## Conclusion

The present study examined how gender interacted with chronic pain in SUD and explored provincial differences in the SUD in a national representative sample. Chronic pain affects men and women differently. Different types of chronic pain are associated with different probabilities of SUD in men and women. This research highlights the importance of recognizing gender differences to inform and improve prevention and treatment efforts. These findings are novel and important as they move the field further toward the understanding of similar and divergent manifestations of physical conditions and psychiatric well-being among men and women.

## Data Availability Statement

Publicly available datasets were analyzed in this study. This data can be found here: The data that support the findings of this study are from the Public Use Microdata File (PUMF) of the Canadian Community Health Survey – Mental Health, Statistics Canada survey #5015. Access to the data is available to bona fide researchers through institutions participating in Statistics Canada Data Liberation Initiative (DLI) including university libraries throughout Canada – see https://www.statcan.gc.ca/eng/dli/dli. Access can also be arrange directly through DLI enquiries at: statcan.maddli-damidd.statcan@canada.ca.

## Ethics Statement

The secondary analysis of anonymized data does not require ethical approval. Participants in the original survey signed an informed consent and voluntarily participated in the survey. The original survey received ethical approval through Statistics Canada procedures. The patients/participants provided their written informed consent to participate in this study.

## Author Contributions

YS and CD: conceived the idea of this study and together with XM drafted the manuscript. YS and XM: conducted the statistical analyses. CD and XM: provided critical feedback toward developing the research question and results interpretation and had been involved in manuscript redrafting. All authors contributed to the revisions of the initial draft of the manuscript and have read and approved the final draft.

## Conflict of Interest

The authors declare that the research was conducted in the absence of any commercial or financial relationships that could be construed as a potential conflict of interest.

## Publisher's Note

All claims expressed in this article are solely those of the authors and do not necessarily represent those of their affiliated organizations, or those of the publisher, the editors and the reviewers. Any product that may be evaluated in this article, or claim that may be made by its manufacturer, is not guaranteed or endorsed by the publisher.
