# Supplementary material for: Does Gender Moderate the Relationship Between Chronic Pain and Substance Use Disorder? Insights From a National Canadian Population Survey
Source: Front Psychiatry. 2022 Mar 2;13:799655. doi: 10.3389/fpsyt.2022.799655 (PMC8924112; doi:10.3389/fpsyt.2022.799655)
Supplement: Supplementary file 1 [file Table_1.DOCX]

**- Table S1.** Correlations among substance use disorder and potential covariates, Community Health

Survey of Mental Health and Well-being-Mental Health Component, Canada, 2012

|  | 1 | 2 | 3 | 4 | 5 | | 6 | 7 |
| --- | --- | --- | --- | --- | --- | --- | --- | --- |
| 1-Substance use disorder | - |  |  |  |  | |  |  |
| 2-Age | 0.14* | - |  |  |  | |  |  |
| 3-Education attainment | -0.06* | -0.20* | - |  |  | |  |  |
| 4- Marital status | 0.07* | 0.48* | -0.10* | - |  | |  |  |
| 5- Household income | -0.08* | -0.23* | 0.34* | -0.12* | - | |  |  |
| 6- Ethnicity | -0.05* | -0.19* | 0.04* | -0.09* | -0.05* | | - |  |
| 7- Immigrant status | -0.06* | 0.01 | -0.06* | -0.03* | 0.04* | | -0.47* | - |
| Note: *P<0.001(2 tailed). | | | | | |  |  |  |
